# Supplementary material for: High-quality Arabidopsis thaliana Genome Assembly with Nanopore and HiFi Long Reads
Source: Genomics Proteomics Bioinformatics. 2021 Sep 3;20(1):4–13. doi: 10.1016/j.gpb.2021.08.003 (PMC9510872; doi:10.1016/j.gpb.2021.08.003)
Supplement: Supplementary Table S2 — Misassembled regions in TAIR10.1 assembly [file mmc2.docx]

**Table S2 Misassembled regions in TAIR10.1 assembly**

| **Chr ID** | **Start** | **End** | **Length (bp)** | **Related protein-coding genes** |
| --- | --- | --- | --- | --- |
| NC_003076.8 | 5,775,535 | 5,776,346 | 812 | AT5G17522 |
| NC_003076.8 | 5,775,586 | 5,777,350 | 1765 | AT5G17523 |

*Note*: These regions are duplicated in Chr 5 assembled from Contig 7 (shown in green in Figure 1) in this study.
