# Supplementary material for: High-quality Arabidopsis thaliana Genome Assembly with Nanopore and HiFi Long Reads
Source: Genomics Proteomics Bioinformatics. 2021 Sep 3;20(1):4–13. doi: 10.1016/j.gpb.2021.08.003 (PMC9510872; doi:10.1016/j.gpb.2021.08.003)
Supplement: Supplementary Table S7 — Classified repeats in Col-XJTU and TARI10.1 assemblies [file mmc7.docx]

**Table S7 Classified repeats in Col-XJTU and TARI10.1 assemblies**

| **Type** | | **Col-XJTU** | **TARI10.1** |
| --- | --- | --- | --- |
| Retroelement | | 10,209 | 3927 |
|  | SINE | 525 | 299 |
|  | LINE | 1366 | 782 |
|  | LTR element | 8318 | 2846 |
| DNA transposon | | 7480 | 4273 |
| Satellite | | 1567 | 791 |
| Simple repeat | | 36,404 | 19,032 |
| Low complexity | | 9032 | 4889 |

*Note*: All annotations were analyzed using the same pipeline described in Materials and methods section. SINE, short interspersed nuclear element; LINE, long interspersed nuclear element; LTR, long terminal repeat element.
